# The Impact of Dengue on Economic Growth: The Case of Southern Taiwan

**DOI:** 10.3390/ijerph17030750

**Published:** 2020-01-24

**Authors:** Chien-Yuan Sher, Ho Ting Wong, Yu-Chun Lin

**Affiliations:** 1 Department of Business Management, National Sun Yat-sen University, Kaohsiung 80424, Taiwan; towel29@gmail.com; 2 Institute of Health Care Management, Department of Business Management, National Sun Yat-sen University, Kaohsiung 80424, Taiwan

**Keywords:** dengue, economic impact, growth accounting, spatial regression, Taiwan

## Abstract

Dengue has long been a public health problem in tropical and subtropical countries. In 2015, a dengue outbreak occurred in Taiwan, where 43,784 cases were reported. This study aims to assess the impact of dengue on Southern Taiwan’s economic growth according to the economic growth model-based regression approach recommended by the World Health Organization (WHO). Herein, annual data from Southern Taiwan on the number of dengue cases, income growth, and demographics from 2010–2015 were analyzed. The percentage of reduction of the average income per capita in 2015 due to the dengue outbreak was estimated. Dengue was determined to have a negative linear economic impact on Southern Taiwan’s economic growth. In particular, a reduction of 0.26% in the average income per capita was estimated in Southern Taiwan due to the 2015 outbreak. If the model is applied alongside other dengue outbreak forecast models, then the forecast for economic reduction due to a future dengue outbreak may also be estimated. Prevention and recovery policies may subsequently be decided upon based on not only the number of dengue cases but also the degree of economic burden resulting from an outbreak.

## 1. Introduction

In the first half of the 20th century, dengue fever was a rare disease with low mortality rates and was considered an unimportant public health problem. Unexpectedly, it started becoming a serious global public health problem in the 1970s, although it was exclusively limited to Southeast Asia during the 1950s and 1960s. The global growth of dengue in 1970 was mainly due to the pace of controlling the disease vector (i.e., *Aedes aegypti*) and the inability to catch up with the epidemic’s expansion [1,2]. Currently, although the problem is especially critical in countries in tropical regions, such as Malaysia [3], Vietnam [4], and Singapore [5], countries in subtropical regions, such as Taiwan [6] and China [7], are not exempt from an outbreak. In recent decades, the global incidence of dengue has grown dramatically according to the World Health Organization (WHO) [8]. It was estimated that the number of dengue cases have more than doubled every decade from 1990–2013. For instance, the number increased sevenfold—from 8.3 million (95% CI: 3.3–17.2) in 1990 to 58.4 million (95% CI: 23.6–121.9) in 2003 [9].

Unfortunately, no specific treatment for dengue fever exists, and the consequences of infection may be fatal. The death rate of severe dengue infection may be more than 20% without early detection and proper medical care [10]. The number of deaths increased around 36.5%—from 8277 deaths (95% CI: 5353–10,649) in 1992 to 11,302 deaths (95% CI: 6790–13,722) in 2010 [9]. Vaccines for dengue were made available in 2015, but WHO solely recommends that the use of vaccines be limited in highly endemic areas defined by a seroprevalence of 70% or higher [8].

Due to dengue’s significantly negative impacts, many research studies have been devoted to this issue from various angles, which include identifying the determinants of dengue’s incidence [11,12], forecasting dengue’s incidence [13,14], and estimating the resulting economic burden [4,15]. Publications on estimating the economic burden associated with dengue are few compared to other dengue-related research works primarily because making such estimations is not easy [16]. Currently, the majority of the available research studies are based on the cost-of-illness methodology, which divides economic impact into direct (i.e., expenses induced by the illness), indirect (i.e., the loss of production value due to the reduced working time induced by the illness) and intangible costs (e.g., the cost of pain and suffering) [17,18]. In particular, Lun et al. [15] analyzed dengue’s economic cost and burden during the epidemic and non-epidemic years in Taiwan from 1998–2014 based on the cost-of-illness methodology. The authors found that 115.3 disability-adjusted life years (DALYs) per million population were annually lost to dengue, and the cost during epidemic years was 12.3 times higher than that during non-epidemic years. Beatue and Vong [19] estimated the cost and burden of dengue based on DALYs and the cost-of-illness methodology for Cambodia, determining that the DALYs ranged from 24.3–100.6 per 100,000 population, and the costs during a large epidemic in 2007 were 4.3 times higher than the overall annual costs in 2008. In 2009, WHO [20] published a guideline which suggested that an economic growth model-based regression approach may be used to assess economic losses. By further comparing the losses in years with and without a dengue outbreak, dengue’s economic impact may then be obtained, although the researchers who have adopted WHO’s recommended approach are few [21,22].

Aside from the abovementioned economic growth model-based regression approach recommended by the WHO, spatial considerations are also meaningful when included in the regression-modeling process and when assessing the economic burden of a dengue outbreak. The spillover effect may serve as an example here [23] in that an increase in the dengue incidence rate of village *j* will directly cause a decrease in its average income growth. This reduction will influence the economic growth of the village *j*’s neighbors, and the change in the growth among *j*’s neighbors will produce another reduction in village *j*’s growth, and so on. Moreover, the decrease in the growth of village *j*’s neighbors will also continue lowering the growth in all villages adjacent to them, and so on, until all the villages linked by a chain of adjacent villages are affected.

Taiwan is an island country with a humid, subtropical climate and close links with other dengue-endemic Southeast Asian countries. The aim of this study is to assess dengue’s impact on economic growth in Southern Taiwan. The assessment followed the WHO guideline using an economic growth model-based regression approach as a supplementary to the assessments using the cost-of-illness approach [15,20]. As spatial effects were also considered, a spatial regression model was adopted rather than the traditional multiple linear regression model.

## 2. Materials and Methods

### 2.1. Conceptual Framework

This study presents a discussion of how dengue cases have affected the economic growth in each village or urban village (i.e., neighborhoods, or 里) in Southern Taiwan. Following the growth accounting literature [24,25], the contribution of various factors to the economic growth in village *j* can be expressed by the equation below:

(1) $$ln\;\left(\frac{Y_{j,t}}{Y_{j,t-1}}\right)\;=\theta_{k}ln\;\left(\frac{K_{j,t}}{K_{j,t-1}}\right)\;+\theta_{E}ln\;\left(\frac{E_{j,t}}{E_{j,t-1}}\right)\;+g_{j,t}^{TFP}$$

where we assume that $\theta_{k}+\theta_{E}=1$, while $\theta_{k}$ and $\theta_{E}$ represent the share of the total input contributed by capital and labor inputs, respectively; $g_{j,t}^{TFP}$ is the growth of total factor productivity (TFP) in village *j* from year *t* − 1 to year *t* and can be further divided into two parts: The first, denoted by $g_{t}^{T}$, is the TFP growth attributable to nationwide events, such as knowledge improvement or institutional change, while the second, denoted by $g_{j,t}^{RE}$ is the change in regional economic efficiency, such as the residents’ level of social contact. Hence, $g_{j,t}^{TFP}=g_{t}^{T}+g_{j,t}^{RE}$.

In Equation (1), *Y_j,t_* and *K_j,t_* indicate the total income and capital input (or initial capital stock) for village *j* in year *t*, respectively. *E_j,t_* is the effective labor input for village *j* in year *t*, which is a function of the quality and quantity of labor input. One can express *E_j,t_* with the equation below:

(2) $$E_{j,t}=Q_{j,t}H_{j,t}L_{j,t}$$

here *Q_j,t_*, *H_j,t_*, and *L_j,t_* denote the workers’ quality, average working hours per worker, and the number of workers, respectively.

By subtracting $ln\left(\frac{L_{j,t}}{L_{j,t-1}}\right)$ from both sides in Equation (1),

(3) $$\begin{array}{c} ln\;\left(\frac{Y_{j,t}}{Y_{j,t-1}}\right)\;-ln\;\left(\frac{L_{j,t}}{L_{j,t-1}}\right)\;\\ =\theta_{k}ln\;\left(\frac{K_{j,t}}{K_{j,t-1}}\right)\;+\theta_{E}ln\;\left(\frac{E_{j,t}}{E_{j,t-1}}\right)\;-ln\;\left(\frac{L_{j,t}}{L_{j,t-1}}\right)\;+g_{j,t}^{TFP}\\ =\theta_{k}ln\;\left(\frac{K_{j,t}}{K_{j,t-1}}\right)\;+\theta_{E}\left(ln\;\left(\frac{Q_{j,t}}{Q_{j,t-1}}\right)\;+ln\;\left(\frac{H_{j,t}}{H_{j,t-1}}\right)\;+ln\;\left(\frac{L_{j,t}}{L_{j,t-1}}\right)\;\right)-ln\;\left(\frac{L_{j,t}}{L_{j,t-1}}\right)\;+g_{t}^{T}+g_{j,t}^{RE} \end{array}$$

Hence,

(4) $$ln\;\left(\frac{\frac{Y_{j,t}}{P_{j,t}}}{\frac{Y_{j,t-1}}{P_{j,t-1}}}\frac{\frac{P_{j,t}}{L_{j,t}}}{\frac{P_{j,t-1}}{L_{j,t-1}}}\right)=\theta_{k}ln\;\left(\frac{K_{j,t}}{K_{j,t-1}}\right)\;+\left(\theta_{E}-1\right)ln\;\left(\frac{L_{j,t}}{L_{j,t-1}}\right)\;+\theta_{E}ln\;\left(\frac{Q_{j,t}}{Q_{j,t-1}}\right)\;+\theta_{E}ln\;\left(\frac{H_{j,t}}{H_{j,t-1}}\right)\;+g_{t}^{T}+g_{j,t}^{RE}$$

where *P_j,t_* is the population of village *j* in year *t*. Because $\theta_{k}+\theta_{E}=1$, Equation (4) becomes

(5) $$\begin{array}{c} g_{j,t}^{y}=ln\;\left(\frac{\frac{Y_{j,t}}{P_{j,t}}}{\frac{Y_{j,t-1}}{P_{j,t-1}}}\right)\;\\ =\theta_{k}ln\;\left(\frac{\frac{K_{j,t}}{L_{j,t}}}{\frac{K_{j,t-1}}{L_{j,t-1}}}\right)\;+\theta_{E}ln\;\left(\frac{Q_{j,t}}{Q_{j,t-1}}\right)\;+\theta_{E}ln\;\left(\frac{H_{j,t}}{H_{j,t-1}}\right)\;+ln\;\left(\frac{\frac{L_{j,t}}{P_{j,t}}}{\frac{L_{j,t-1}}{P_{j,t-1}}}\right)\;+g_{t}^{T}+g_{j,t}^{RE}\\ =\theta_{k}g_{j,t}^{k}+\theta_{E}g_{j,t}^{Q}+\theta_{E}ln\;\left(\frac{H_{j,t}}{H_{j,t-1}}\right)\;+\emptyset_{j,t}+g_{t}^{T}+g_{j,t}^{RE} \end{array}$$

where $g_{j,t}^{y}$, $\;g_{j,t}^{k}$ and $g_{j,t}^{Q}$ are the growth of average income, the change of the initial stock of capital per worker, and the change in the workers’ quality, respectively; $\emptyset_{j,t}$ denotes the change in the ratio of workers to the population.

Dengue’s impact on $g_{j,t}^{y}$ can be identified from two aspects. First, dengue can result in a decline in village *j*’s working hours. Let *n*, *nl*, and *d_j_* represent the average health worker’s working hours, the average decline in working hours resulting from dengue, and the number of dengue cases in village *j*, respectively; thus, we get the following:

(6) $$H_{j,t}=\frac{n\left(L_{j,t}-d_{j,t}\right)+\left(n-nl\right)d_{j,t}}{L_{j,t}}=n\left(1-\frac{nl}{n}\frac{d_{j,t}}{L_{j,t}}\right)$$

let *γ* be *nl*/*n*. The numerators of *γ* and $\frac{d_{j,t}}{L_{j,t}}$ are the average decline in working hours resulting from dengue and the number of dengue cases in village *j*, respectively; both of them have a small value because only 6.37% of the villages have the number of dengue cases to the working age population over 1% (Table 1). Hence, the product of *γ* and $\frac{d_{j,t}}{L_{j,t}}$ (i.e., *γ(d_j,t_/L_j,t_)*) is a miniscule number. Thus,

(7) $$ln\;\left(\frac{H_{j,t}}{H_{j,t-1}}\right)\;=ln\;\left(\frac{1-\gamma \frac{d_{j,t}}{L_{j,t}}}{1-\gamma \frac{d_{j,t-1}}{L_{j,t}-1}}\right)\;\approx -\gamma \left(\frac{d_{j,t}}{L_{j,t}}-\frac{d_{j,t-1}}{L_{j,t-1}}\right)=-\gamma g_{j,t}^{d}$$

where $g_{j,t}^{d}$ is the change in the ratio of dengue cases to the number of workers in village *j*.

Secondly, a dengue outbreak can lower the level of social contact and damage regional economic efficiency [20]. Assuming that the damage dengue inflicts upon regional economic efficiency is related to the ratio of dengue cases to workers, $g_{j,t}^{RE}$ can be broken down into *u_j,t_* (i.e., the change resulting from other factors) and $-\delta g_{j,t}^{d}$ (i.e., the change resulting from the differences between epidemics).

In summary, we can transform Equation (5) into

(8) $$g_{j,t}^{y}=\theta_{k}g_{j,t}^{k}+\theta_{E}g_{j,t}^{Q}+\emptyset_{j,t}+g_{t}^{T}-\left(\theta_{E}\gamma +\delta \right)g_{j,t}^{d}+u_{j,t}$$

Our goal is to estimate the value of (*θ_E_γ* + *δ*) using cross-village panel data.

### 2.2. Regression Specification

Following the conceptual framework, the regression specification is structured as follows:

(9) $$\begin{array}{c} g_{j,t}^{y}=\beta_{0}+\beta_{1}g_{j,t}^{d}+\beta_{2}g_{j,t}^{Q}+\beta_{3}\emptyset_{j,t}+\alpha_{1}I^{Y}+\alpha_{2}I^{D}+\rho_{1}\overset{J}{\underset{i=1}{\sum }}w_{i}^{j}g_{i,t}^{y}+\pi_{j}+u_{j,t},\\ u_{j,t}=\rho_{2}\sum_{i=1}^{J}w_{i}^{j}u_{i,t}+\varepsilon_{j,t} \end{array}$$

where *I^Y^* is a vector containing year-fixed effects and $\alpha_{1}I^{Y}$ captures the influence of $g_{t}^{T}$ in different years; *I^D^* is a vector containing district-fixed effects. Because no capital stock statistics are available at the village level, we assume that $g_{j,t}^{k}$ is similar for villages in the same district (e.g., one district may have 10–80 villages), while we apply *α_2_I^D^* to determine the influence of $g_{j,t}^{k}$ on the economic growth in each village. Moreover, it is reasonable to believe the investment decision made prior to the beginning of year *t* is unrelated to the number of dengue cases that occur in that year. Hence, the lack of detailed information about $g_{j,t}^{k}$ should not result in a biased estimation of its impact.

In Equation (9), $w_{i}^{j}$ is an element of a spatial weighting matrix (J×J), while *J* is the number of villages. If villages *j* and *i* are neighbors, then 1 ≥ *w^j^_i_* > 0; otherwise, $w_{i}^{j}$ = 0, and moreover, $w_{i}^{j}$ = 0. In this study, if *j* and *i* share the same border or even a mere vertex, then they are defined as neighbors (e.g., queen contiguity-based weights). By this specification, the economic growth and regional economic efficiency in each village can be affected by their neighbors’ performance; *π_j_* is the unobservable panel-level effect, which is independent and identically distributed (i.i.d.) across panels; *u_j,t_* is a disturbance that is i.i.d. across panels and time. Hence, Equation (9) is a spatial autoregressive model with spatially autoregressive errors and random effects. The coefficients in Equation (9) can be estimated by the maximum likelihood estimator derived by Lee and Yu [26].

### 2.3. Data

#### 2.3.1. Study Period

All data dated from 2010–2015 were employed, which represents the time span during which two epidemics occurred in Southern Taiwan (i.e., one each in 2014 and 2015). The time span started in 2010 due to a considerable change in the administrative divisions in that year, wherein old Kaohsiung City merged with old Kaohsiung County and old Tainan City merged with old Tainan County.

#### 2.3.2. Spatial Coverage of the Study

**Figure 1:**
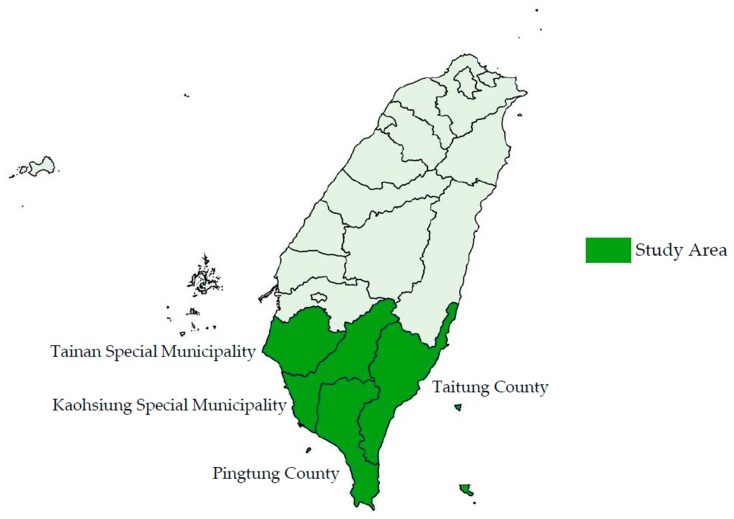
A map of Taiwan.

This study concentrates on dengue’s impact on the villages of Tainan Special Municipality, Kaohsiung Special Municipality, Pingtung County, and Taitung County in Southern Taiwan (Figure 1), all of which are located in the South of the Tropic of Cancer. Only southern Taiwan was chosen for analysis because the physical and economic environment is relatively suitable for dengue transmission and quite different from Northern Taiwan. For example, Kaohsiung is economically less well developed and has relatively hot weather compared to Taipei in Northern Taiwan. Within the study area, we further removed the observations from mountain indigenous districts and small islands (e.g., Green Island, Orchid Island, and Liuqiu Island). The main vector for transmitting dengue is Aedes aegypti, whose distribution in Taiwan around 2010 was mainly in the areas below 1000 m of the four municipalities or counties, excluding the three small islands [6,27]. Hence, outside the study area, only sparse dengue cases were confirmed [6]. Moreover, the economic development pattern in mountain indigenous districts or small islands is different from that in other areas. Including mountain indigenous districts and small islands, which have different development patterns and merely a few dengue cases, would cause a biased estimation on dengue’s impact on economic growth and was hence excluded.

#### 2.3.3. Data Type and Sources

This study focuses on three types of data at the village level: The annual number of dengue cases, annual income growth, and demographic data. The annual confirmed number of dengue cases in each village was calculated using daily confirmed dengue cases with coordinate information, which was provided by the Center for Diseases Control of the Ministry of Health and Welfare in Taiwan [28]. The annual growth of average income in each village was calculated using the mean annual taxable income in each village, to which we applied data from the Fiscal Information Agency, Ministry of Finance.

The demographic characteristics for each village can be found on the website operated by the Ministry of the Interior [29]. Because no labor statistics are available at the village level, a change in the working-age population ratio (i.e., those aged 15–64 years) was applied as a proxy for the change in the ratio of workers to the population. The percentage of the population aged 15–64 years who possessed a bachelor’s degree or higher, which is noted by *C_j,t_*, measured workers’ quality; specifically, *Q_j,t_* = 1 + *C_j,t_*. Hence, if village *j* houses no working-age villagers with a bachelor’s degree or higher, then *Q_j,t_* = 1, whereas if all working-age villagers in *j* hold a bachelor’s degree or higher, then *Q_j,t_* = 2. The descriptions of all variables are provided in Table 2.

When estimating a spatial autoregressive model, the boundaries of all the villages must be recorded such that each village’s neighbors can be defined. The map of Taiwan’s village-level administrative divisions can be found at the Taiwan Geospatial Information website, which is operated by the National Development Council [30]; the map version to which we referred in this study was that from 2015.

## 3. Results and Discussion

### 3.1. Descriptive Statistics

These four municipalities and counties have a total of 2254 villages; after excluding the villages from the mountain indigenous districts and the small islands, we still included 2147 villages in the sample. The descriptive statistics of the main variables for these villages from 2010–2015 are also presented in Table 2.

**Figure 2:**
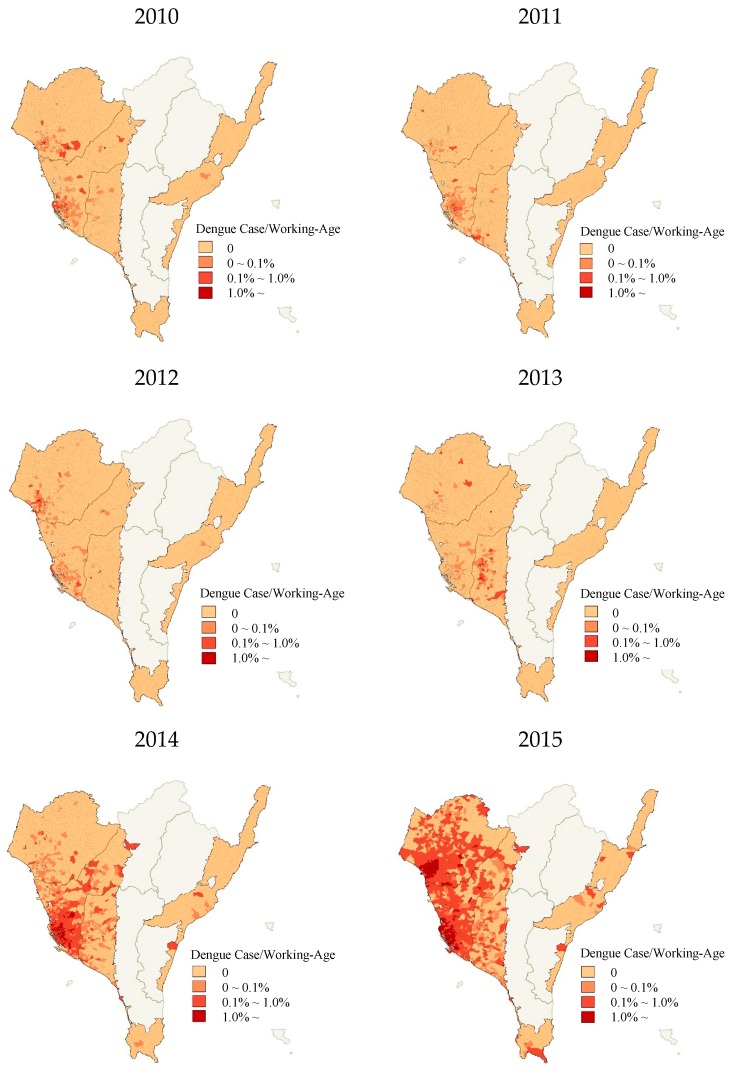
The ratio of confirmed dengue cases to the working-age population in each village from 2010–2015.

In Table 2 and Figure 2, one may observe that the number of dengue cases in each village varied across time and village from 2010–2015; the mean was 4.92, whereas the standard deviation was 18.82. The ratio of dengue cases to the working-age population for each village was also calculated, for which the distribution is illustrated in Figure 2. Again, this ratio greatly varied across time and village. In 2010 and 2012, with the exception of a few villages in old Tainan City and Kaohsiung City, this ratio for most villages did not exceed 1%. In 2011, 10 villages with a ratio over 1% were sparsely located in old Tainan City, old Kaohsiung City, and Pingtung County. In 2013, nine villages with a ratio over 1% were all located in Pingtung County. However, in 2014, the ratio of 226 villages exceeded 1%, all of which were located in Kaohsiung Special Municipality. In 2015, a large outbreak resulted in the ratio of 555 villages exceeding 1%, distributed among Tainan Special Municipality, Kaohsiung Special Municipality, and Pingtung County.

The mean annual growth of income per capita was also calculated (i.e., $g_{j,t}^{y}$) for observations with a similar ratio of dengue cases to the working-age population, for which the result is provided in Table 1. Among 12,882 observations (2147 villages over 6 years), 8948 exhibited no dengue cases, and the average income growth for these observations was 2.48%. For 821 observations with a ratio over 1%, the average income growth was merely 0.52%. Observations with a higher ratio also had a roughly lower annual income growth, which is possibly some evidence that $g_{j,t}^{y}$ was reduced as a result of the dengue outbreak.

### 3.2. Estimation Results

In this section, the spatial autoregressive model described as Equation (9) was estimated. A squared term of the change in the ratio of dengue cases to the working-age population (i.e., $g_{j,t}^{d}$) was added in the regression model to identify the possible nonlinear relationship between the dengue illness and economic growth, for which the results are offered in Table 3.

In Table 3, one may observe that the estimates for the spatial lags of the average income growth ($\rho_{1}$ = 0.507) and the covariate lags of the disturbance ($\rho_{2}$ = −0.495) were both statistically significant (*p* < 0.01), which implies that a village’s economic growth and regional economic efficiency affected those factors among its neighbors. One may also find that the estimated coefficient for the independent variable $g_{j,t}^{d}$ was negatively and statistically significant (−0.226, *p* < 0.05), while the estimated coefficient for the independent variable $g_{j,t}^{d}$^2^ was insignificant (1.641, *p* > 0.05). These estimates may together suggest that dengue has a negative linear impact on economic growth, although its total impact on growth cannot simply be calculated using the estimated coefficients for $g_{j,t}^{d}$ and $g_{j,t}^{d}$^2^ because a dependent-variable lag is present in the model.

The calculation of dengue’s total impact from the recursive process of the spillover effect, which was mentioned in the introduction section, was based on the estimates in Table 3 as well as the spatial weighting matrix that defines neighborhood relationships among villages, for which Table 4 illustrates the results. In this table, the average impact on each village’s growth was calculated in the event that an incremental change in $g_{j,t}^{d}$ or $g_{j,t}^{d}$^2^ occurred in all the villages. The direct effect is the impact from the incremental change within the village after the recursive process, whereas the indirect effect is the impact from the incremental changes from the other villages after this recursion. Thus, the total effect is the sum of the direct and indirect effects [31].

In Table 4, the average of the total effect of an incremental change in $g_{j,t}^{d}$ is presented as statistically significant (−0.407; *p* < 0.05), while that in $g_{j,t}^{d}$^2^ does not statistically differ significantly from zero (2.950; *p* > 0.05). These two numbers together suggest an incremental change’s negative linear marginal effect on the increased number of dengue cases. Presuming all the villages had a mean value of $g_{j,t}^{d}$ (i.e., $g_{j,t}^{d}$ = 0.0015) in year *t*; these two numbers suggest that if the $g_{j,t}^{d}$ values for all the villages become 0.0025 (e.g., $g_{j,t}^{d}$ values increase by 0.001; i.e., following Equation (7), the number of dengue cases in a village increase by 0.001*L_j,t_ in year *t*)—all else being equal—then the average growth of income per capita in a village would decrease by approximately 0.040% ((−0.407+2×2.950×0.0015)×0.001).

We can apply another method to understand the meaning of our estimated results. Table 5 depicts the calculated marginal means and the predicted means of growth, where every observation is treated as though its $g_{j,t}^{d}$ (i.e., “the change in the ratio DengueCase/working-age population” in Table 5) had been equal to the numbers listed in Table 5 but the values of other independent variables had remained the same. The mean of the annual growth of income per capita for all the observations (displayed in Table 2) was 0.0234, while the mean of the $g_{j,t}^{d}$ values was 0.0015. If $g_{j,t}^{d}$ for each observation had become 0.0025, then the predicted mean of growth would have been 0.0230; in other words, the marginal mean would have been lower than the real mean by 0.04%. Moreover, if $g_{j,t}^{d}$ for each observation had become 0.0099 (mean plus one standard deviation), then the predicted mean of growth would have been 0.0200.

A large dengue outbreak occurred in 2015; the mean of the $g_{j,t}^{d}$ values for the observations held in 2015 was 0.0065, while the mean growth of income per capita for the observations held in the same year was −0.0034. If the ratio of dengue cases to the working-age population in 2015 had been identical to that in 2014 among all the villages (i.e., the $g_{j,2005}^{d}$ values had been 0), then the predicted mean of 2015’s growth would have been −0.0008. The 0.26% reduction in the average growth is our estimation regarding the impact of that large outbreak on the growth in Southern Taiwan in 2015 (Table 5).

### 3.3. Comparison to Similar Studies Using the Cost-of-Illness Approach

This study presented a compilation of an economic impact estimation for the 2015 dengue outbreak in Southern Taiwan and produced a regression model to relate the growth of average income (i.e., $g_{j,t}^{y}$) with the change in the ratio of dengue cases to the number of working age population (i.e., $g_{j,t}^{d}$) and other controlling variables (i.e., Equation (9)). Based on the regression model, different hypothetical scenarios can be tested through plugging in suitable values in the model’s independent variables. In contrast, Luh et al.’s study [15] only calculated the exact values of the yearly disease burden (i.e., DALY), the direct cost (i.e., medical cost), and the indirect cost (i.e., lost workdays and caregiver fees) for the period from 1998–2014 [15]. As the calculation of the above three costs is not based on a parametric model, calculation for hypothetical cases is difficult because the calculation has to be restarted from the beginning.

Despite the effort of calculating the economic impacts, the cost-of-illness approach adopted in different studies [4,15,19] is also difficult for economists to interpret the estimated impacts. This is because the first type of cost estimated by the cost-of-illness approach (i.e., disease burden) only reflects the amount of time, activity, or ability that is lost by an individual as a result of dengue-induced death or disability. The second and third types of cost (i.e., direct and indirect costs) only reflect the cost that the study area needs to pay because of a dengue outbreak in terms of monetary aspects. It is difficult to determine if these costs are high or low to a study area because its capability to bear the cost is also important to make the conclusion. The area of disaster management also has a similar concept suggesting that a country can stand with disasters if its resilience to disasters is strong enough [32]. Although many studies have adopted the cost-of-illness approach to compare the costs in the years with and without dengue outbreak [15,19], the interpretation of the cost is still a difficult task, and hence it is difficult to compare the results of different studies. In contrast, the outcome of this research (i.e., the percentage of income growth reduction) is a relative scale, so it is more suitable to be used to make the comparison. In addition, the outcome also provides a possibility for researchers to compare the economic impact of a dengue outbreak within a single study area with other events, such as a financial crisis, which could be a future research direction of this study. The comparison is feasible because the percentage of income growth reduction is not specifically designed for any event, whereas the three costs (i.e., DALY, medical costs, and caregiver fees) in the cost-of-illness approach are obviously only applicable in health-related events, but not in a financial crisis.

### 3.4. The Weakness of the Economic Growth Model-Based Regression Approach

Although it is claimed that the economic growth model-based regression approach is recommended by WHO [20], related research studies using the same approach are limited [21,22]. Currently, the cost-of-illness approach remains the most popular choice among researchers conducting multi-country comparison studies, but what researchers have done in systematic review studies [2,9] is not easy. Nevertheless, as the economic growth model-based regression approach is recommended by WHO [20], it will become more popular after a certain period. At that time, enough results will be available for conducting multi-country comparisons on the economic impacts of dengue outbreaks.

## 4. Conclusions

This study is the first to offer an analysis of dengue’s impact on economic growth in Southern Taiwan using the economic growth model-based spatial regression approach recommended by WHO. As expected, the final result demonstrated that dengue has a negative linear economic impact on Southern Taiwan’s economic growth, and it also establishes the spatial autoregressive nature of that impact. In particular, when information on the 2015 dengue outbreak in Southern Taiwan was entered into the economic growth model, a reduction of 0.26% in the average income per capita was estimated. The model is not solely able to estimate the economic impact of dengue retrospectively; in fact, when the economic growth model is applied alongside other dengue outbreak forecast models [13,14], the forecast of economic reduction due to a future dengue outbreak can additionally be predicted. For example, imagine that a dengue outbreak forecast model predicted an outbreak in the future along with the expected number of dengue cases. The economic growth model could be used to assess the economic burden of this predicted outbreak based on the expected number of dengue cases obtained from the forecast model. In this way, prevention and recovery policies may then be decided upon based on the number of dengue cases and the degree of economic burden inflicted by a dengue outbreak.

## Figures and Tables

**Table 1:** The average annual income growth per capita for observations with different number of dengue cases to the working-age population ratio (n = 12,882).

Types	Number of Observations	Average Growth (%) ^a^
Observations with the ratio of dengue cases to the working-age population equals zero	8948	2.48
Observations with the ratio of dengue cases to the working-age population from 0 to 0.1%	1301	2.97
Observations with the ratio of dengue cases to the working-age population from 0.1% to 1%	1812	2.06
Observations with the ratio of dengue cases to the working-age population over 1%	821	0.52
All observations	12,882	2.34

^a^ We observe 2147 villages across 6 years and calculate the mean for the growth of income per capita for observations with a similar ratio of dengue cases to working age population.

**Table 2:** Description of dependent and explanatory variables (n = 12,882 observations) ^a^.

Variables	Description	Mean	S.D.
**Dependent Variable**			
$g_{j,t}^{y}$	The annual growth of average income	0.0234	0.0692
**Explanatory Variable**			
$d_{j,t}$	The annual confirmed number of dengue cases in village *j* and year t	4.9190	18.8159
$g_{j,t}^{d}$	The change in the ratio (DengueCase/working age population) from year *t* − 1 to year *t* in village *j*	0.0015	0.0084
*C_j,t_*	The percentage of population, aged 15 to 64, with a bachelor’s degree or higher in village *j* and year t	0.2989	0.1098
$g_{j,t}^{Q}$	The annual growth of (1 + *C_j,t_*) in village *j* and year t	0.0082	0.0085
$L_{j,t}/P_{j,t}$	The ratio of working age population (those aged 15 to 64) to total population in village *j* and year t	0.8332	0.0537
$\emptyset_{j,t}$	The change in the ratio of working age population to the total population in village *j* and year t	−0.0037	0.0086
District	A vector of dummy variables denoting the district where the village is located	--	--
Year	A vector of dummy variables denoting the year of observation points	--	--

^a^ The descriptive statistics are calculated using the observations of 2147 villages from 2010–2015 within Tainan Special Municipality, Kaohsiung Special Municipality, Pingtung County and Taitung County.

**Table 3:** Result of the spatial regression analysis on the effects of dengue illness on the annual average income growth per capita (N = 12,882 observations) ^a^.

Independent Variables	Coeff.	S.E.
$g_{j,t}^{d}$	−0.226	(0.100) *
$g_{j,t}^{d}$ ^2^	1.641	(1.095)
$g_{j,t}^{Q}$	0.237	(0.062) **
$\emptyset_{j,t}$	0.530	(0.068) **
$\rho_{1}$	0.507	(0.030) **
$\rho_{2}$	−0.495	(0.046) **
Constant	0.012	(0.002) **
Controlled by yearly dummies ^b^	Yes	
Controlled by district dummies ^b^	Yes	
Pseudo R^2^	0.146	

Note: Dependent variable: The annual growth of average income per capita in each village. ^a^ We analyze the impact of dengue on annual income growth in 2147 villages from 2010–2015 using spatial regression. ^b^ We have five yearly dummies for 6 years and 106 district dummies for 107 districts. * *p* < 0.05; ** *p* < 0.01.

**Table 4:** The direct, indirect, and total effects of dengue illness (N = 12,882) ^a^.

	$g_{j,t}^{d}$		$g_{j,t}^{d}{}^{2}$	
	dy/dx	S.E.	dy/dx	S.E.
Direct effect	−0.235	(0.104) *	1.705	(1.137)
Indirect effect	−0.172	(0.077) *	1.245	(0.841)
Total effect	−0.407	(0.180) *	2.950	(1.972)

^a^ The direct, indirect, and total effects are calculated using the estimates in Table 3. * *p* < 0.05.

**Table 5:** Marginal means under different values in the main dependent variable ^a^.

The Change in the Ratio (DengueCase/Working Age Population)	Marginal Mean	S.E.	n
For all observations (2010–2015) ^b^			
0	0.0240	(0.0007)	12882
0.0005	0.0238	(0.0007)	12882
0.0015	0.0234	(0.0007)	12882
0.0025	0.0230	(0.0007)	12882
0.0099	0.0200	(0.0017)	12882
For observations in 2015 ^b^			
0	−0.0008	(0.0021)	2147
0.0015	−0.0014	(0.0019)	2147

^a^ Marginal means are the predicted means of the growth of income per capita in case the change in the ratio (DengueCase/working age population) equals the numbers listed in Table 5. ^b^ The average of the growth of income per capita for all observations is 0.0234, and the average of the growth for observations in 2015 is −0.0034.
